# Influence of Copper-Strontium Co-Doping on Bioactivity, Cytotoxicity and Antibacterial Activity of Mesoporous Bioactive Glass

**DOI:** 10.3390/gels8110743

**Published:** 2022-11-16

**Authors:** Akrity Anand, Susanta Sengupta, Hana Kaňková, Anna Švančárková, Ana M. Beltrán, Dušan Galusek, Aldo R. Boccaccini, Dagmar Galusková

**Affiliations:** 1 Centre for Functional and Surface Functionalized Glass, Alexander Dubček University of Trenčín, 911 50 Trenčín, Slovakia; 2 Institute of Biomaterials, University of Erlangen-Nuremberg, 91058 Erlangen, Germany; 3 Departamento de Ingenieria y Ciencia de los Materiales y del Transporte, Escuela Politécnica Superior, Universidad de Sevilla, 41011 Seville, Spain

**Keywords:** mesoporous glass, co-doping, bioactivity, cytotoxicity, antibacterial activity

## Abstract

Mesoporous bioactive glass (MBG) is an extensively studied biomaterial used for the healing of bone defects. Its biological applications can be tailored by introducing metallic ions, such as strontium (Sr) and copper (Cu), which can enhance its functionalities, including osteogenetic, angiogenetic and antibacterial functionalities. In this study, Cu and Sr ions were co-doped (ratio 1:1) with x = 0.5, 1 and 2 mol% each in glass with an intended nominal composition of 80SiO_2_-(15-2x)CaO-5P_2_O_5_-xCuO-xSrO and synthesized with an evaporation-induced self-assembly (EISA)-based sol-gel technique. XRD confirmed the amorphous nature of the glass, while compositional analysis using ICP-OES confirmed the presence of dopant ions with the required amounts. A TEM study of the MBG powders showed fringes that corresponded to the formation of a highly ordered mesoporous structure. The Cu-Sr-doped MBG showed a positive effect on apatite formation when immersed in SBF, although the release of Cu and Sr ions was relatively slow for 1 mol% of each co-dopant, which signified a stable network structure in the glass. The impact of the Cu and Sr ions on the osteoblast-like cell line MG-63 was assessed. At the particle concentrations of 1 wt./vol.% or lower, the cell viability was above 50%. An antibacterial test was conducted against Gram-negative *E. coli* and Gram-positive *S. aureus* bacteria. With a sequential increase in the co-doped ion content in the glass, the zone of inhibition for bacteria increased. The results suggest that the doping of MBG with Cu and Sr ions at up to 2 mol% can result in tailored sustained release of ions to enhance the applicability of the studied glass as a functional biomaterial for bone regeneration applications.

## 1. Introduction

Mesoporous bioactive glass (MBG) is a third-generation biomaterial widely known for its controlled mesoporous texture and ability to bond with living bone tissues, providing a smart platform for drug delivery and bone regeneration applications [1,2,3,4,5]. The main component of MBG is a tertiary glass structure with the composition SiO_2_-CaO-P_2_O_5_, and the leaching of silica (Si), calcium (Ca) and phosphorus (P) in body fluid directly participates in the bone-forming activity. Si favors metabolic processes, such as the formation and calcification of osseous tissues, while Ca and P assist in the formation of bone minerals, such as hydroxyapatite Ca_10_(PO_4_)_6_(OH)_2_), and are a prerequisite in bone formation and resorption [6] Recently, a new strategy has been formulated for the composition of MBG involving the incorporation of specific metallic (therapeutic) ions to enhance the biological impact and functionality of the material. Several trace elements, such as strontium (Sr), copper (Cu), zinc (Zn) and magnesium (Mg), are present in the human body and show anabolic effects during bone metabolism. To imitate the human bone tissues, MBG with enhanced bioactivity is conjugated with the desired metallic ions. The release of these therapeutic ions from BG is believed to improve the angiogenesis, growth and mineralization of bone tissues [7,8].

Among these therapeutic ions, copper (Cu) is an important trace element that is present between 1.5 and 2.1 mg per kg of the human body and is required for the proper functions of several proteins/enzymes [9,10]. The presence of Cu stimulates endothelial cell proliferation and promotes angiogenesis in vitro [11,12]. Moreover, Cu exhibits antibacterial activity, as it can bond with the thiol, imidazole, carboxylic and amine functional groups of microbial proteins and change their structure, which increases the permeability of the cell membrane, eventually leading to its collapse and loss of function [13]. The release of modifier ions (Ca^2+^), along with Cu^2+^, from the Cu-doped bioactive glass, increases the pH in the physiological fluid, which inhibits the growth and inactivates the enzymes or proteins of bacteria [14].

Wu et al. prepared Cu (0, 1, 2 and 5 mol.%) containing 80SiO_2_-15CaO-5P_2_O_5_ MBG scaffolds using P123 surfactant and polyurethane sponges. The Cu-doped MBG scaffolds were found to stimulate hypoxia-inducible factor 1-alpha (HIF-1a) and vascular endothelial growth factor (VEGF) expression in human bone marrow stromal cells (hBMSCs). The scaffold promoted bone-related gene expression, such as alkaline phosphatase, osteocalcin and osteopontin, which significantly improved the osteogenic differentiation of hBMSCs [15]. Cu doping in MBG facilitates antibacterial activity against *E. coli*, *S. aureus* and *S. epidermidis* and stimulates osteogenesis and angiogenesis [16,17].

Another trace element naturally present in the human body is strontium, and nearly 98% of Sr is localized in osseous tissue. In a 70 kg human body, the total amount of Sr is around 0.32 g. The physical and chemical properties of strontium are analogous to those of calcium as they belong to the same group and have similar ionic radii (Ca^2+^ 1Å and Sr^2+^ 1.16Å) [18,19]. Sr can assemble in bone by interchanging with Ca in the crystal lattice of hydroxyapatite and has been widely studied for bone regeneration. Sr ions help in osteoblast replication and inhibit osteoclast activity. They can even enhance osteogenic differentiation and help in stabilizing bone structure [20,21]. Wang et al. developed hierarchically mesoporous and microporous 80SiO_2_-15CaO-5P_2_O_5_ MBG scaffolds using P123 surfactant and polyurethane sponges as co-templates and doped them with 0.75, 1.5 and 2.25 mol.% of SrO in substitution for CaO. The proliferation of mesenchymal stem cells on the MBG scaffolds was enhanced with the higher amount of Sr dopant (2.25% SrO) after 7 and 14 days of culture compared with lower concentrations of dopant [22]. [. Wu et al. synthesized 80SiO_2_-15CaO-5P_2_O_5_ MBG for periodontal tissue engineering applications and doped it with Sr (2.5, 5 and 10 mol.%) by replacing SiO_2_. The increasing amount of Sr ions could disrupt the ordered orientation of (SiO_4_)^4−^ at the time of the self-assembly reaction, which resulted in potential structural defects in the atomic array and significantly altered the mesopore structure [23]. In the literature, several studies can be found on the individual doping of Cu and Sr ions, and the strategy for co-doping has been reported previously.

Very few investigations have been undertaken on multifunctional Cu/Sr co-doped MBG systems. Bari et al. developed cetyltrimethylammonium bromide (CTAB) based Cu-Sr containing MBG in the SiO-CaO binary system, which resulted in the spherical morphology of the particles [24] while Balasubramanian et al. used a pluronic surfactant to develop 60SiO_2_-(36-x)CaO-4P_2_O_5_-xCuO-xSrO MBG system using EISA techniques [25]. The surfactant molecules self-assemble themselves into micelle structures (spherical or cylindrical shape) during the EISA process. The micelles hydrophilic parts face outside and are in contact with the components of bioactive glass (Si, Ca and P, etc.), while the hydrophobic parts face inside, towards the core of the micelle. When the mixture is dried, and the surfactant is removed, a bioactive glass with a well-ordered mesoporous structure is formed [26]. However, the study of Balasubramanian et al. reveals the absence of ordered mesoporous structure in the system and the formation of a low surface area between 155–210 m^2^/g. In the present study, Cu and Sr as co-dopant ions were added for the first time in 80SiO_2_-(15-2x) CaO-5P_2_O_5_ -xCuO-xSrO mol.% MBG system and parameter x assumes the values of 0.5, 1 and 2 mol.%. CaO acts as a network modifier, and the abundance of Ca in the system decreases the network connectivity of silica. In addition, the higher Ca content glass results in a higher exchange of Ca ions from glass and H_3_O^+^ ions from the SBF, leading to excessive glass reactivity and an increase in local pH. Therefore, a lower amount of glass modifiers (CaO up to 15 mol.%) has been added to the present system to control the glass reactivity.

A highly ordered mesoporous structure with a high surface area was obtained for Cu-Sr doped MBG using P123 surfactant. The addition of CuO and SrO in the present system enhances the overall textural properties compared to its controlled glass without doping. The Cu-Sr co-substituted glass is studied in terms of its amorphous phase, composition, microstructure, bioactivity, cytotoxicity towards osteoblasts-like cells and antibacterial activity. It is hypothesized that co-doping with Cu and Sr in the present MBG system can provide combined functionalities for bone regeneration, as Cu can enhance angiogenesis and antibacterial activity while Sr can promote osteogenic properties. Thus, these selected cations would offer multifunctional effects in MBG and act as a promising material for bone tissue regeneration.

## 2. Results and Discussion

### 2.1. Physiochemical and Textural Characterisations of MBG

**Figure 1:**
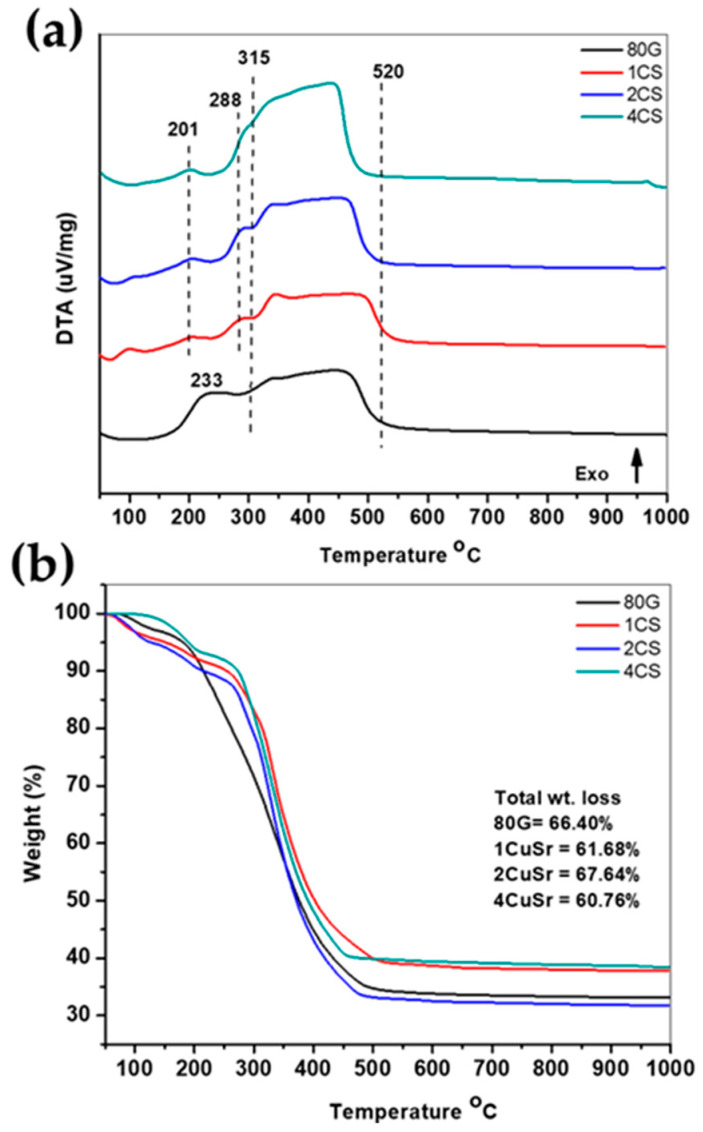
DTA (**a**) and TGA (**b**) plots of as-prepared base glass (80G) and co-doped CS-MBG (1CS, 2CS, 4CS) powders.

The DTA-TGA thermogram of all as-prepared MBG powders, i.e., the base glass 80G and co-doped CS-MBG (1CS, 2CS and 4CS), was carried out to optimize the temperature for the calcination of MBG powders. The sol-gel-based EISA process was carried out in a chamber with controlled humidity (~75%) and a surrounding temperature of 32 ± 3 °C. In very humid conditions in the chamber, BG sols transformed from liquid to gel structure due to the hydrolysis followed by the condensation of TEOS molecules. Additionally, to provide a mesoporous texture inside the glass, the surfactant has also been used, which may result in additional weight loss. The DTA-TGA plots of co-doped CS-MBG and base glass 80G are shown in Figure 1a,b. Three similar patterns of exothermic peaks were formed for all CS-MBG at temperatures 201 °C, 288 °C and a broad peak at 315 to 520 °C while the base glass 80G showed two exothermic events at temperature 233 °C and an exothermic hump from 288 to 520 °C. The peaks below 201 °C are related to the loss of adsorbed water molecules from the glass surface, which corresponds to the weight loss of around 6–8% for co-doped MBG. The base glass showed a slight shift in the peak around 233 °C, which is attributed to the loss of volatile compounds in water molecules. A small hump of the exothermic peak at 288 °C for co-doped powders may represent the onset of the decomposition of organic moieties. A high amount of weight loss (up to 50–56%) was observed for all the MBG from temperature 315–520 °C, probably due to nitrates and surfactant loss [27]. The combustion of the surfactant may liberate a high amount of CO_2_ and H_2_O molecules as products which eventually results in a broad exothermic peak. The theoretical density of CaO is 3.4 g/cm^3^ is much lower than the density of CuO (6.31 g/cm^3^) and SrO (4.7 g/cm^3^) so, probably when Ca is replaced by Cu and Sr in MBG composition (up to 2 mol.%), the density of the overall system increases. The increased density may result in higher weight loss in 2CS in comparison with other co-doped systems [28]. The total weight loss of the prepared MBG up to 600 °C is depicted in the TGA plot. The prepared base and co-doped glass lost all organic components of the precursors and structure-directing agents at around 600 °C. Thus, all the as-prepared MBG (doped and co-doped) were calcined at 700 °C for 5 h with a heating rate of 2 °C/min. The MBG powders calcined at 700 °C were used afterwards throughout the study.

**Figure 2:**
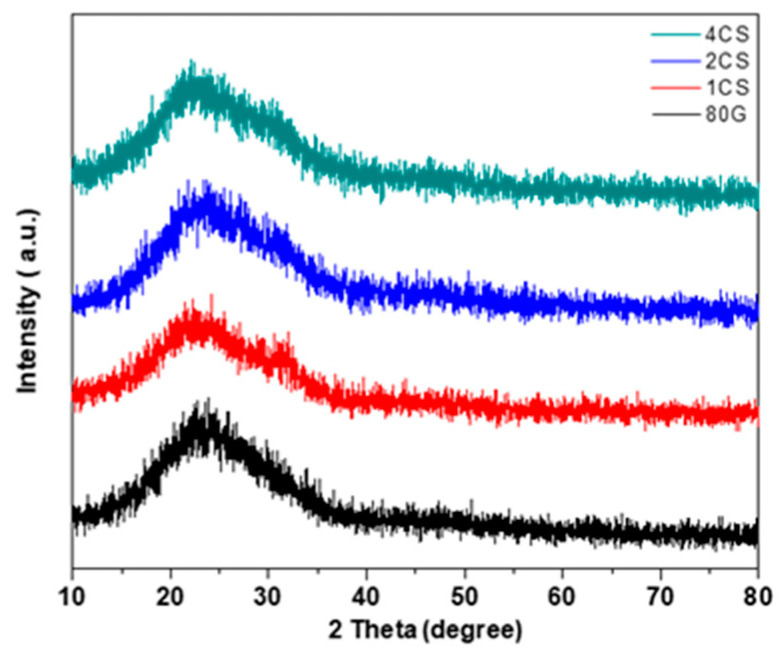
Represents the XRD patterns of the base and CS-MBG powders calcined at 700 °C.

The compositional analysis of the MBG powders was undertaken by ICP-OES, as depicted in Table 1 and Figure 2, with a standard deviation. The dissolution of silica-based glass takes place in the presence of HF due to the breaking up of strong Si–O bonds and the formation of hexafluorosilicic acid, which is present in water in a dissociated form (SiF_6_^2−^) even at a temperature of <35 °C. As all the prepared MBG have a high content of SiO_2_ (80 mol.%), there is a real possibility of potential loss of Si during decomposition due to the partial formation of volatile silicon tetrafluoride. Thus, recovery for silicon analysis cannot be fully achieved by this method and values for the amount of SiO_2_ present in prepared MBG were calculated through normalization to 100%. The ICP-OES analysis confirms the presence of co-doped ions, although the actual compositions of glasses vary from the resultant glass compositions, especially the amount of phosphorus is significantly lower compared to the nominal value for all prepared glasses. The lower amount of P_2_O_5_ in the MBG system is possibly due to the occurrence of phase transition from solid to liquid state during calcination. The calcination of all MBG powders was performed at 700 °C, and the boiling temperature of P_2_O_5_ was 360 °C; therefore, there is a high chance for P_2_O_5_ decomposition in the system. The lower amount of P_2_O_5_ remains in the glass system as feasibly P_2_O_5_ acts as a network former, and it may form a bond with the silica and calcium during glass formation.

The diffusion halos at around 15–35° of 2θ document the presence of an amorphous phase in prepared glasses. The formation of a broad diffuse peak at this 2θ range is characteristic of silicate glasses [29].

**Figure 3:**
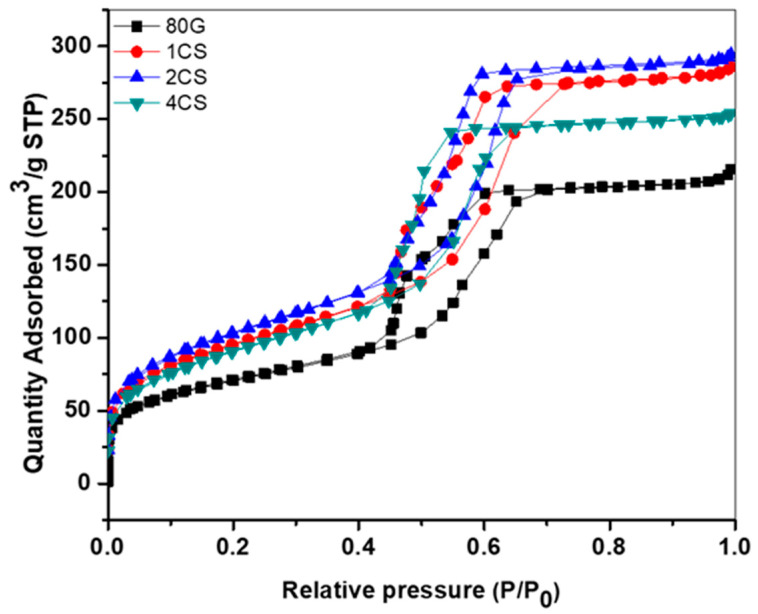
N_2_ adsorption–desorption isotherm plots of base glass and co-doped MBG.

The process of the adsorption–desorption of nitrogen gas by the pores of the MBG provides information about the surface area pores structure and the shape of the adsorption–desorption isotherm. The base glass and CS-MBG are characterized by the adsorption–desorption Type IV isotherm, which is shown in Figure 3. A complete lack of microporosity from the relative pressure (P/P_0_) from 0 to 0.4 and an increase in adsorption volume from 0.4 to 0.7 P/P_0_ that later reached a plateau (saturation point) was observed. The two adsorption and desorption curves are nearly vertical and parallel to each other at this (0.4 to 0.7 P/P_0_) range of nitrogen gas uptake. This distinct loop of adsorption and desorption represents Type H1 hysteresis, which is consistent with the findings of Lopez-Noriega et. al, and signifies the formation of narrow distribution of uniform cylindrical mesopores open at both ends for all the MBG powders [30,31]. Table 2 shows the specific surface area, pore size and total pore volume of all MBG powders. The 80G glass shows a surface area of 253 m^2^/g, while with the addition of co-dopant ions (Cu and Sr), the surface area of all MBG powders increased gradually. The 2 mol.% of CS has a relatively high surface area of 378 m^2^/g, in comparison to 1CS (348 m^2^/g) and 4CS (332 m^2^/g) powders. Due to the increase in the ionic concentration of bivalent ions, the self-assembly of surfactant molecules can be disturbed, and the involvement of a number of surfactant molecules in one template is reduced. This may lead to smaller but a higher number of template sites. Therefore, after calcination, a higher number of pores with smaller pore diameters will form, leading to a higher specific surface area of the materials for a higher amount of co-doping. The 80G glass has a large pore size of 5.2 nm in comparison to other CS glass, which confers the possibility of a larger pore size corresponding to a lower surface area. Sample 2CS showed a larger surface area but also a slightly large pore size (4.80 nm) in comparison to the 4CS pore size (4.71 nm), probably due to the larger number of pores present in 4CS particles. Similar results were obtained by J. Jiménez-Holguín et al. for their Cu-containing MBG, where surface area and pore volume increased with the increasing percentage of CuO (0 to 5%) in the system [32]. The pore volume of MBG is directly related to the surface area. The pore volume of 80G glass is relatively low (up to 0.33 cm^3^/g) in comparison to all co-doped CS-MBG. From the study, it is assumed that the addition of co-dopant ions up to 2 mol.% in the MBG system forms a stable glass structure network and shows a positive effect on the material properties, such as increasing the surface area and pore volume of the material.

**Figure 4:**
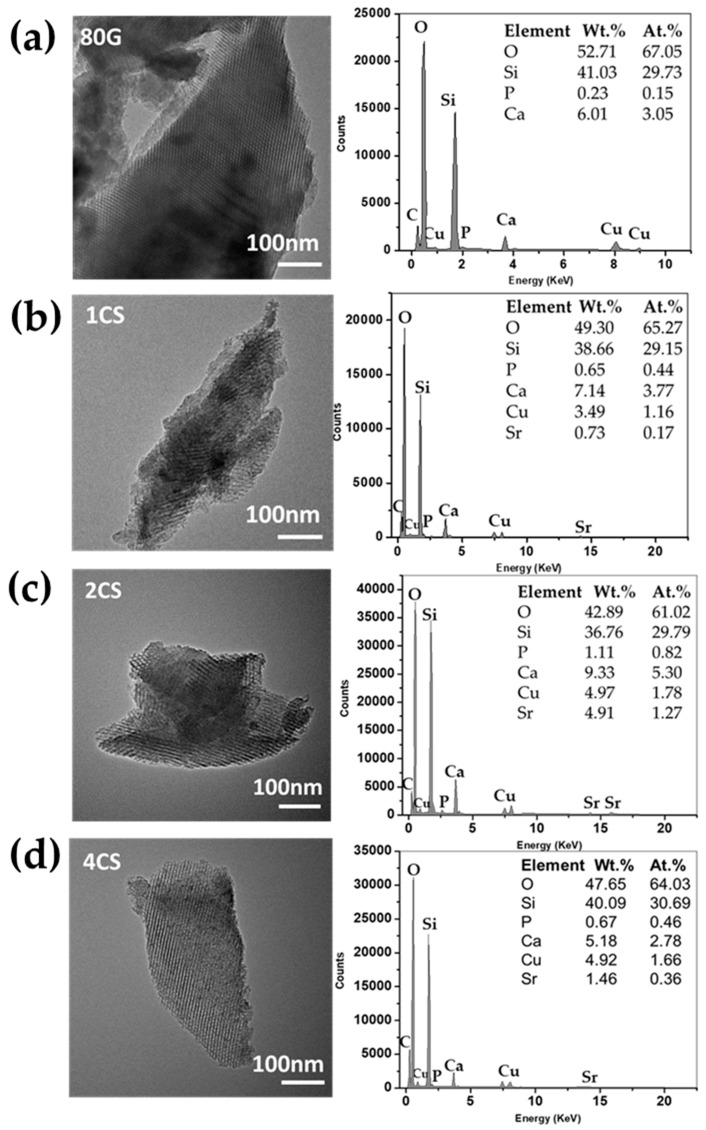
TEM images with EDX analysis of MBG powders. (**a**) 80G, (**b**) 1CS, (**c**) 2CS and (**d**) 4CS2.2. In vitro Bioactivity Assessment.

The formation of uniform cylindrical-like mesoporous texture was further confirmed by TEM examination (Figure 4). The TEM images show fringes that represent the formation of a highly ordered mesoporous structure in all the MBG. The formation of ordered mesoporous structures is mainly due to regular packing (self-assembly) of micelles at the nanometer range followed by homogenous distribution of organic–inorganic species that developed homogenized textural properties in MBG after calcination [33]. In the present study, the molar ratio of precursor solution and surfactant molecule was constant for all prepared MBG. This optimized amount of surfactant results in homogenized inorganic–organic pairs, and no additional surfactant species were left to interrupt the formation of ordered mesoporous structure. Moreover, the condensation of inorganic species through EISA techniques by controlling the humidity and temperature results in organized meso-texture inside all MBG powders [34]. From the TEM images, it is confirmed that the introduction of co-dopant ions in the base glass composition did not disrupt the textural properties of prepared MBG. Moreover, the textural properties, especially the microstructure of the BGs, play a significant role in nucleation and growth, followed by crystallization of the apatite layer. In the case of MBG, the formation of the apatite layer might be improved because of the higher surface area, pore volume and pore size of MBG. It facilitated better interaction of biological fluids with the surface of the glass and, consequently, increased the leaching of therapeutic ions in the surrounding medium [5,35]. Herein all the co-doped MBG showed uniform mesopores orderliness of 4.7–5.0 nm and a higher surface area (up to 378 m^2^/g) in comparison to the base 80G glass (253 m^2^/g). The textural properties of CS-MBG were expected to improve the in vitro bioactivity significantly.

### 2.2. In Vitro Bioactivity Assessment

#### 2.2.1. Ions Release Profile and pH Change

**Figure 5:**
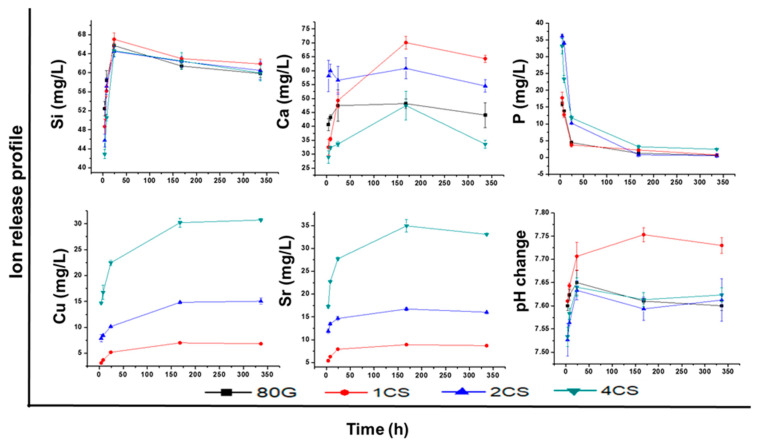
Release profile Si, Ca, P, Cu and Sr ions (mg/L) in SBF and pH measurements for base glass (80G) and co-doped MBG (1CS, 2CS and 4CS).

With in vitro conditions, the release of calcium and phosphorus from the MBG samples is expected to trigger the formation of a predominantly hydroxyapatite layer on its surface and, under in vivo conditions to form a bond with living bone tissues. The leaching of Si, Ca, P, Sr, and Cu ions from MBG and the change in the pH value were studied in SBF (Figure 5). When less durable glass is soaked in SBF, rapid cationic (Na^+^ or Ca^2+^) exchange occurs with H_3_O^+^ ions from the SBF, leading to an increase in hydroxyl ions concentration resulting in a pH of the media higher than 7.4 [36]. An increase in pH up to 24 h (measured at a temperature of 37 °C), possibly due to intense ionic exchange between the MBG and SBF solution, was observed for all of the co-doped MBG samples as well for 80G. The release profile of silicon (Figure 5), a network former in MBG glass, shows the degradation of co-doped MBG predominantly in the first 24 h, then the release of ions decelerates for the remaining time interval of 14 days. The hydroxyl ion concentration measured after 7 and 14 days stabilizes, and the pH level of 7.8 is not exceeded. Simultaneously with ion exchange, the breaking of Si-O-Si (network former) bonds due to the interaction of hydroxyl ions (OH^−^) might occur. Silicon released in the SBF is present as Si(OH)_4_ complex while Si-OH (silanols) remain at the glass-SBF interface. Later these silanol groups form a silica-rich layer, which might help the formation of an amorphous CaO-P_2_O_5_ layer on the MBG surface.

The plotted concentration of leached Ca^2+^ in the graph includes subtraction from the initial calcium concentration in the SBF solution. Unlike the 1CS sample, a steady increase in Ca^2+^ ions had been detected in the monitored time intervals for all of the MBG systems. Despite the lower amount of phosphorus determined by ICP-OES (<1 mol.% of P_2_O_5_) in all of the prepared, co-doped MBG, phosphate phase formation was likely initiated. Starting from 4 h, a continuous decrease in the phosphorus concentration was detected. The amount of Ca released in SBF was sufficient to destabilize the solution equilibrium and contribute to new phase formation precipitated from the solution. A drastic drop in PO_4_^3−^ concentration was observed within 24 h of soaking, which dropped further for 7 days and almost disappeared from the SBF at 14 days. This phenomenon may indicate the continuous formation of an amorphous apatite layer on the MBG. The decrease in Ca ions after 7 days might indicate that there should be transformation of the apatite layer from amorphous to crystalline, which could be further confirmed from FTIR and XRD analysis in the following discussions. A lower release of Ca^2+^ has been observed for 80G and 4CS compared with other MBG due to the lower specific surface area and Ca amount in the respective glass system.

Along with calcium and phosphorus, the destabilization of the MBG structure facilitated the liberation of other important therapeutic ions. The concentration of leached modifier ions (Sr^2+^ and Cu^2+^) in SBF increases in time up to 7 days, and then the concentration of dissolved ions remains unchanged after 14 days. It was assumed that the release of co-dopant ions would be high for the high content of the co-doped MBG system. A similar amount of ions were released from the Sr-doped MBG system investigated by Wu et al. [5]. Overall, for each system, the Sr release rate was slightly higher than Cu, probably due to the larger ionic radii of the Sr ions (1.16 Å) in comparison to the Cu (0.73 Å) ions.

Despite the highest pH values being measured for the 1CS samples up to 7 days, probably referred to the increased concentration of Ca^2+^ in the solution, the pH values of the SBF solution for the entire bioactivity assay were <7.8. The ideal value of pH 7.0–7.6 for osteoblasts activity was reported in [37,38].

#### 2.2.2. Bioactivity on MBG Surfaces

**Figure 6:**
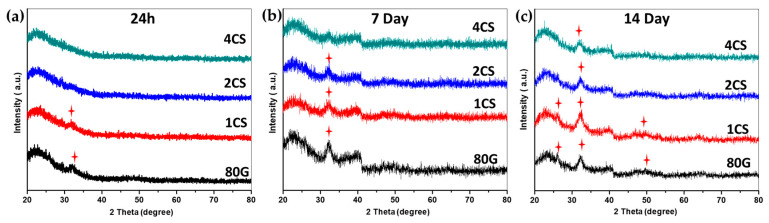
XRD patterns of SBF soaked MBG powders after (**a**) 24 h, (**b**) 7 days, and (**c**) 14 days; (red star marks indicate possible formation of a new hydroxyapatite (HAp) phase.

The formation of the Ca-P layer over the surface of the MBG powders was initially characterized by XRD, and the results are presented in Figure 6. All of the glasses showed an amorphous nature before immersion in SBF. After 24 h of soaking in SBF low-intensity diffuse diffraction maxima were detected for the base glass 80G and co-doped glass 1CS at 2θ 32.2°, while 2CS and 4CS were XRD amorphous. After 7 days of immersion, the base glass 80G and co-doped 1CS and 2CS glass showed a formation of diffraction maximum at 2θ = 32.2°, while 4CS glass only had a broad hump at this 2θ value. With 14 days of immersion, MBG glasses showed a progressive characteristic diffraction pattern of HAp. Typical diffraction maxima at 2θ: 26°, 32.2°, and 49.6° found for 80G and 1CS indicate the formation of the HAp layer [39]. However, with the extension of the soaking time (up to 14 days), 2CS and 4CS showed the formation of a more pronounced maximum at 32.2°. As the XRD analysis was conducted using the powder-based technique, the appearance of the corresponding diffraction maxima completely depends on the amount of the samples and crystals present in the sample [40] ]. Therefore, XRD analysis only provides the probable phases of the crystalline phosphate matrix, which could be further verified by subsequent FTIR analysis.

**Figure 7:**
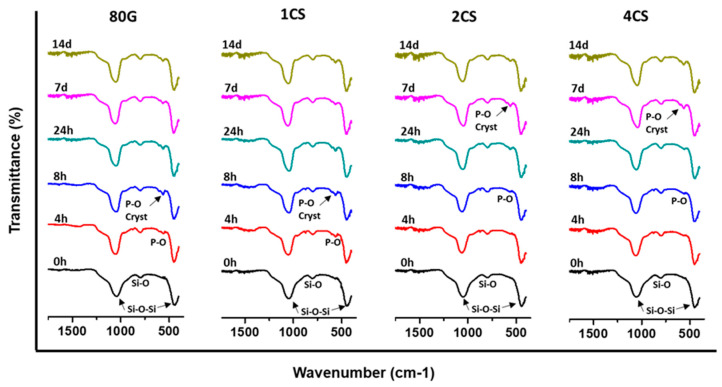
FTIR spectra of all MBG powders before and after 0 h, 4 h, 8 h, 24 h, 7 d and 14 d of immersion in SBF.

The FTIR spectra were measured on MBG powders before soaking and after soaking in SBF for all the time periods, i.e., 4 h, 8 h, 24 h, 7 days and 14 days, as plotted in Figure 7. Before immersion, all of the powders showed only common bands at around 1040–1070 cm^−1^ and at 440–460 cm^−1^ which represent asymmetric bending vibration Si-O-Si bonds and the band at 800–810 cm^−1^ which signifies the symmetric stretch of Si-O bonds [41]. After 4 h of soaking the glass, 80G and 1CS showed a new peak at 560 cm^−1^, which could be attributed to the vibration of P-O bonds in an amorphous phosphate phase, while 2CS and 4CS glass showed the formation of a phosphate phase after 8 h of soaking. The band at 560 cm^−1^ splits into two bands at 560 and 600 cm^−1^ assigned to the crystalline phosphate phase observed for 80G and 1CS after 8 h of soaking, while 2CS and 4CS showed the splitting of the band after 7 days of SBF soaking. Nevertheless, all the co-doped and bare MBG showed the bands attributed to crystalline PO_4_^3−^ (in line with XRD analysis Figure 6) representing HAp layer formation on the surfaces [30].

**Figure 8:**
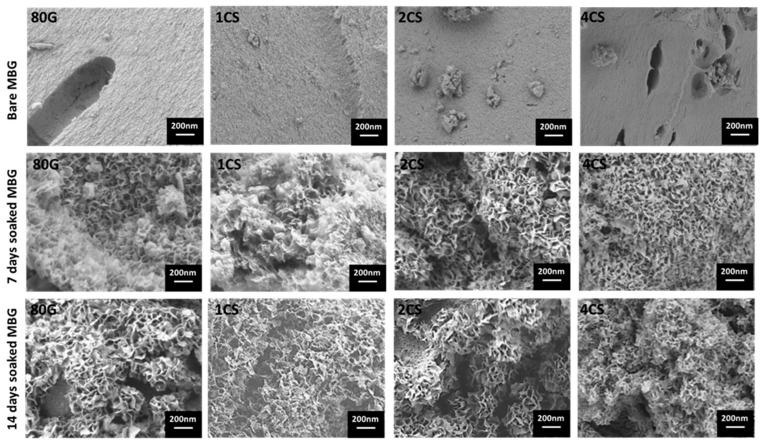
FESEM images of bare MBG powders and after SBF soaked (7 and 14 days) MBG powders.

The formation of a crystalline phosphate layer for 80G and co-doped powders before (bare powder) and after soaking in SBF for 7 and 14 days is documented in Figure 8. The change in the glass morphology, especially in terms of the new apatite layer deposition, could be observed for all glasses. A cauliflower-like morphology, characteristic of HAp crystals, was observed for all of the powders after 7 days. The co-doping of Cu and Sr ions in the MBG structure did not affect the HAp formation. However, from the FTIR spectra, it was seen that the 80G glass and lowest co-doped system (1CS) showed a crystalline HAp layer only after 8 h of SBF soaking. The addition of dopant ions up to 4 mol.% positively contributes to the HAp formation after 7 days of SBF soaking.

### 2.3. In Vitro Cytotoxicity Assessment

**Figure 9:**
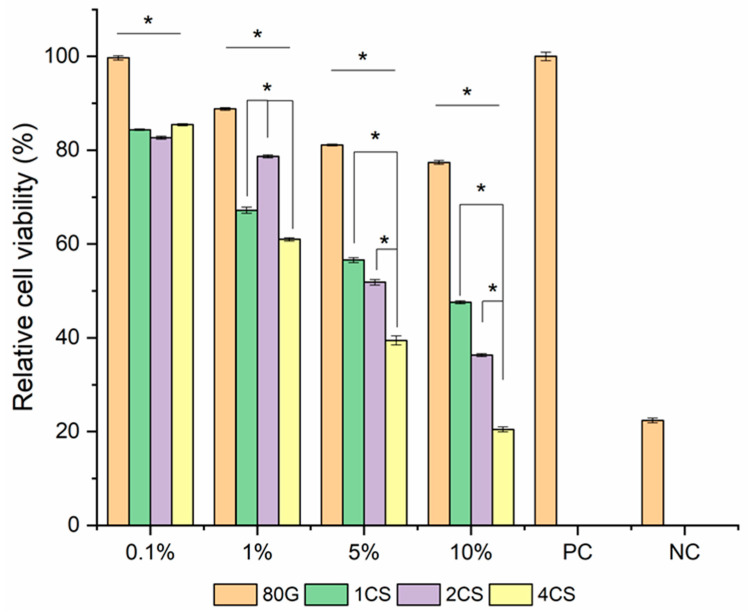
Relative cell viability percentage of MG-63 cells cultured with 0.1, 1, 5 and 10 wt./vol.% extract of MBG (n = 9, PC = positive control, NC = Negative control, samples in triplicate, * *p* < 0.05).

The percent of MG-63 cell viability cultured with the different concentrations of MBG powders in DMEM are presented in Figure 9. The biocompatibility assay was based on the release of ions from MBG in media for 24 h at 37 °C according to International Standard Organization (ISO/EN 10993-5) protocol (International Organization for 1992) [42]. The supernatant was collected, diluted (at different concentrations viz.,10, 5, 1 and 0.1 wt. /vol.%), and then incubated with cells for 48 h to evaluate the vitality or mortality of the cells. The viability of MG-63 cells only with DMEM media is used as the positive control, and MG-63 cells in 6% vol. of DMSO is used as the negative control [43]. In general, after 48 h of culture the viability of the MG-63 cells decreased by 10% wt. /vol.% MBG extracts medium and increased with higher dilution.

The base 80G glass prepared using Si, Ca and P only, unlike co-doped MBG, showed a higher cell viability percentage (≥77%), even in 10 wt./vol.% extract medium. The cell viability of the co-doped glasses was relatively less (≤50%), and the lowest was observed for 4CS. Copper is present in the 4CS sample in the highest amounts, so its sudden release in the first hours is, therefore, considerably higher (Figure 5) compared to other co-doped MBG samples and might explain lower cell proliferation. The presence of therapeutic ions in a concentrated solution could be toxic, and this has been reported previously in several studies [44,45]. The gradual increase in cell viability was noticed with the serial dilution of extracts from the MBG for all of the glass systems. Nevertheless, 1% ionic extracts from 2CS MBG showed the highest cell viability compared with the other co-doped glass systems. It could happen at this dilution for the 2CS glass system that the ionic concentration is optimum for a high cell proliferation rate. With the highest dilution (0.1%) for all of the MBG systems, there was no significant change in cell viability. At that dilution point, all the co-doped MBG showed cell viability above 80% and also biocompatibility, according to previous studies [45,46] (Modglin, Brown et al. 2013, Balasubramanian, Hupa et al. 2017, Kurtuldu, Mutlu et al. 2021). For the 5 and 10 wt./vol.% extract, a high amount of Cu and Sr release could cause negative effects on MG-63 cells and their viability.

In general, when human osteoblast cells are cultured on bioactive glasses, they produce an extracellular matrix, which mineralizes to form bone nodules without any usual supplements of hormones present in the culture medium [37]. Basically, the dissolution of soluble Si and Ca ions from the glasses stimulates osteoblast cell division, the production of extracellular matrix (ECM) proteins and growth factors. The 1CS co-doped MBG showed overall less toxicity compared to other doped glasses, so it may help in the proliferation of osteoblast cells [47,48].

A similar glass system was studied by Balasubramanian et al., which reported the cytotoxicity effect cultured with ST-2 cells with 0.1 wt./vol. % of diluted MBG. However, they also found that even when cell viability is low, it can induce some biological functions, such as the secretion of VEGF [25].

**Figure 10:**
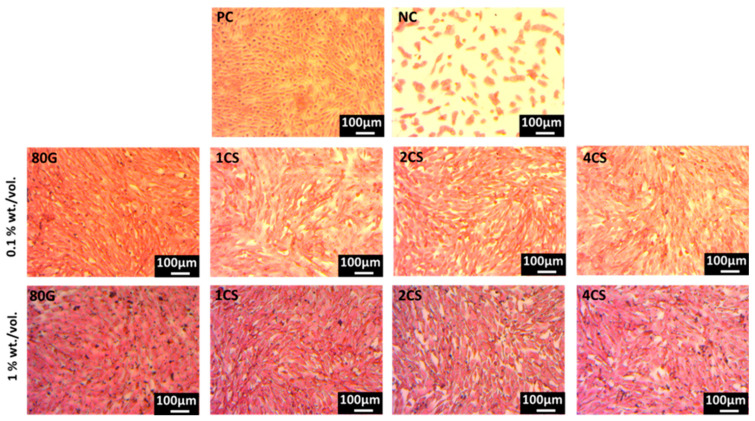
H&E-stained images of MG-63 cells cultured with 0.1 and 1 wt./vol.% extract of MBG along with positive control (PC) and negative control (NC).

Figure 10 shows the light microscopy images of H&E-stained MG-63 cells cultured with 0.1 and 1 wt./vol% extract of MBG in DMEM. The microscopy images confirmed that the cells’ morphology varied from oval to spindle-shaped, and the cells were confluent for the positive control and all MBG except for the negative control. Hence, the H&E-stained images showed a cell morphology that correlated with the WST cell viability measurements.

### 2.4. In Vitro Antibacterial Assessment

The antibacterial test was performed on the MBG pellets using Gram-positive *S. aureus* and Gram-negative *E. coli* bacteria by employing the agar disk diffusion method. The incorporation of Cu in the MBG is a strategy for increasing the inherent antimicrobial effect as an extra functionality in the glass system. According to the standard SNV 195920-1992 protocol, the material can be considered antibacterial if the zone of inhibition is greater than 1 mm [41]. Cu ions have the potential to generate hydroxyl radicals and stimulate the production of reactive oxygen species (ROS), which initially disturb the cellular membrane of bacteria and damage the DNA, enzymes and proteins of the microbes [49]. It is also possible that the release of ions(Ca^2+^ and Cu^2+^) from Cu-doped bioactive glass increases the overall pH in the physiological fluid, which impedes the growth of bacteria and exerts antibacterial properties [14].

**Figure 11:**
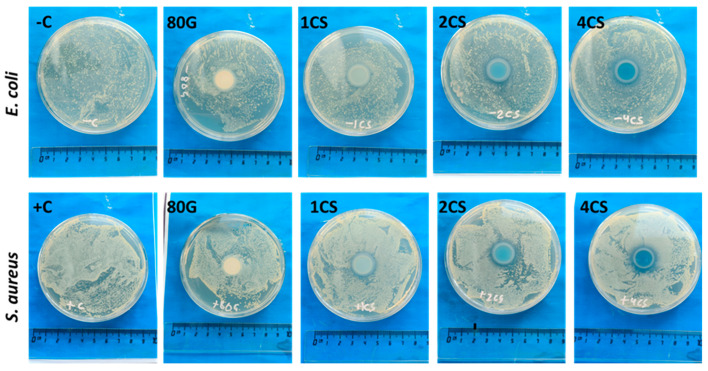
Photographic images of MBG pellets treated with Gram-negative (*E. coli*) and Gram-positive (*S. aureus*) bacteria using the agar disk diffusion method.

Figure 11 represents the photographic images of the Gram-negative control (−C), Gram-positive control (+C) and different MBG pellets (80G, 1CS, 2CS, 4CS) after 24 h of incubation against the antibacterial activity. The base glass 80G is unable to form a zone of inhibition for the bacteria, although, for the co-doped system with a sequential increase in the concentration of Cu, the antibacterial activity was more prominent. The 1CS system showed a small zone of inhibition around 1.5–2 mm, while 2CS pellets showed a moderate zone of inhibition around 2.5 mm, and 4CS (containing 2 mol.% of Cu) showed a considerable zone of inhibition of 3.5 mm against both *E. coli* and *S. aureus* bacteria. Table 3 summarizes the diameters of the zones of inhibition for the MBG samples towards *E. coli* and *S. aureus* bacteria. The test was performed on solid agar media, so the release of Cu ions to show its antimicrobial characteristics is slightly different in comparison to using liquid broth media. Overall, 4CS showed a more considerable inhibition zone for *S. aureus* bacteria than *E. coli* bacteria.

## 3. Conclusions

The 80G base glass and CS-MBG were synthesized using sol-gel-based EISA techniques. Although the amount of P_2_O_5_ content in all MBG was low based on their nominal compositions, a phosphate layer was found on the surface of all of the prepared systems. The formation of an apatite layer was relatively fast (within 8 h of SBF soaking) for the base glass 80G and the moderate co-dopant system (Cu, Sr 1 mol% each). A highly ordered mesoporous structure was formed in the MBG systems, and co-doping with Cu and Sr ions improved the microstructural properties, leading to a higher specific surface area for the co-doped MBG systems. A moderate concentration of co-dopants (Cu and Sr 1 mol% each) showed the highest specific surface area of the MBG, and a sustained, controlled release of therapeutic ions was observed from the material practically from the first day. The release of copper in the first hours did not negatively influence the cytotoxicity and showed that the ionic extracts from MBG up to 1 wt./vol. % are not toxic, and the viability of the MG-63 cell line was above 60%. Furthermore, the zone of inhibitions (up to 2 mm) for Gram-negative and Gram-positive microbes during the antimicrobial study was detected for this MBG system. To conclude, the higher cell viability observed for the 1CS system compared to the other studied co-doped MBG implies that varying amounts of Cu and Sr ions as co-dopants up to 2 mol.% (ratio 1:1) in MBG could be beneficial for bone tissue regeneration alongside the antibacterial properties.

## 4. Materials and Methods

### 4.1. Materials

MBG with a composition of 80SiO_2_-(15-2xCaO)-5P_2_O_5_-xCuO-xSrO mol.%, where x = 0.5, 1 and 2 mol.% was synthesized using pluronic surfactant P123 by the sol-gel method through the evaporation-induced self-assembly (EISA) technique. All the chemical reagents procured were of analytical grade. Tetraethyl orthosilicate [TEOS] (~98.0%, Merck, Germany), calcium nitrate tetrahydrate [Ca(NO_3_)_2_·4H_2_O] (98–99%, VWR International GmbH, Pennsylvania, United States) and triethyl phosphate [TEP] (~99.8%,) were used as the source of Si, Ca and P. Strontium nitrate anhydrous Sr(NO_3_)_2_ (~98%, Alfa Aesar, MA, USA) and Copper(II) chloride CuCl_2_ (~99%, Sigma Aldrich, Darmstadt, Germany) were used as a precursor for Sr and Cu doping ions in the MBG system. Poly(ethylene glycol)-poly(propylene glycol)-poly(ethylene glycol) (PEG-PPG-PEG), Pluronic^®^ P-123 (Mn-5, 800, Sigma Aldrich, Germany) surfactant was used for mesoporous structure formation.

### 4.2. Preparation of Mesoporous Bioactive Glass

In a typical synthesis of 80SiO_2_-15CaO-5P_2_O_5_ MBG, 4.2 g of P123 was dissolved in 63 g of ethanol with 1.05 g of 0.5M HCl. Further, 7.56 g of tetraethyl orthosilicate (TEOS), 0.82 g triethyl phosphate (TEP) and 1.60 g of Ca(NO_3_)_2_·4H_2_O were added under continuous stirring of 1 h intervals and stirred at room temperature for 24 h. The resulting sol was introduced into a Petri dish (~30mL of sol placed in a 100 mm × 20 mm size Petri dish) to undergo evaporation-induced self-assembly (EISA) process with a surrounding temperature of ~32 °C and humidity of ~75%. After 35 h of incubation, the gels were aged for 7 to 9 days in the petri dish at the same temperature. The dried gel was calcined at 700 °C for 5 h with a heating rate of 2 °C/min to obtain the final MBG products. The MBG powder obtained by this process was henceforth termed 80G. Furthermore, for the co-doped system, CuO and SrO were incorporated into the MBG system by replacing CaO in 80SiO_2_-(15-2xCaO)-5P_2_O_5_ -xCuO-xSrO. When x = 0.5 %, 1.50 g of calcium salts and after 1 h of stirring, 0.03 g of copper chloride (CuCl_2_) and 0.048 g of strontium nitrate (Sr(NO_3_)_2_) were added within a 10 min interval, it was henceforth termed 1CS. The compositions of base glass and co-doped glasses with their respective doping percentage is depicted in Table 4.

### 4.3. MBG Powder Characterization

The thermal analysis of the as-prepared MBG powders was conducted using differential thermal–thermogravimetric analysis (DTA-TGA; STA 449F1, Netzsch, Selb, Germany). The analysis was carried out up to 1000 °C at a heating rate of 10 K/min in the air atmosphere using alumina crucibles. Powder X-ray diffraction (XRD) analysis was performed using an X-ray diffractometer (Miniflex 600) equipped with monochromatic Cu- Kα radiation and operated at 40 kV and 15 mA in the 2θ range of 10–80°. A step size of 0.010° and a speed of 2° per min. were used for each measurement. The chemical compositions of MBG were obtained by optical emission spectrometry with inductively coupled plasma (ICP-OES; Agilent 5100 SVDV, Sta. Clara, USA) using 0.55 L/min of carrier gas flow and radiofrequency power of 1.2 kW. The sample was prepared by digesting the MBG powders in a mixture of the following mineral acids: 6 mL hydrochloric acid (HCL; 30%, Merck, Germany), 2 mL nitric acid (HNO_3_; 67–69%, Analytika Ltd., Prague, Czech Republic) and 0.5 mL hydrofluoric acid (HF; 47%, VWRchemicals, Germany) at room temperature. The specific surface area, pore volume and pore size distribution of all MBG were carried out using the nitrogen adsorption–desorption method with an isotherm at 77 K using the Belsorp-mini II analyzer (Microtrac, Osaka, Japan). The surface area of the powders was calculated using the Brunauer–Emmet–Teller (BET) method, and the pore size was obtained using the Barrett–Joyner–Halenda (BJH) method. The formation of a controlled mesoporous structure in all of the MBG was analyzed using (scanning) transmission electron microscopy ((S)TEM and Energy-dispersive X-ray spectroscopy (EDX) using the Talos F200X system, ThermoFisher Scientific, Waltham, MA, USA) with an accelerating voltage of 200 kV. The sample preparation of TEM was carried out by dispersing ~15 mg MBG powders in 10 mL of ethanol and ultrasonication for 10–20 min. The suspension of MBG was then drop cast on top of a holey-carbon-coated copper grid and dried overnight.

### 4.4. In Vitro Bioactivity Assessment

The ability of the MBG powders to form a hydroxyapatite (HAp) layer on their surfaces was analyzed using simulated body fluid (SBF) by following Kokubo’s method [50]. Then, 30 mg of the MBG powders were soaked in 20 mL of SBF for 4 h, 8 h, 24 h, 7 days and 14 days and placed in an incubator at a temperature of 37 °C with an agitation speed of 120 rpm. After each incubation time point, the supernatant SBF was filtered and kept for ion release study using an ICP-OES, and the collected MBG powders were washed with deionized water and ethanol and thereafter dried at room temperature to analyze the apatite layer formation using XRD, FTIR and FESEM. The change in the pH value was also recorded before and after each time interval using a digital pH meter (SevenEasy Plus FP20 benchtop pH/mV micro kit, Mettler Toledo, Columbus, Ohio, USA)

### 4.5. In Vitro Cytotoxicity Assessment

The in vitro cytotoxicity evaluation of all of the MBG powders was carried out using a human osteoblast-like cell line (MG-63 ECACCS, Sigma Aldrich, Darmstadt, Germany). MG-63 cells show functional characteristics of the pre-osteoblastic phase and are successfully used as a model for bone-related research, such as cell–material interactions, adhesion and proliferation [46].

The MG-63 cells were cultured in Dulbecco’s modified eagle medium (DMEM, Gibco Paisley, UK) supplemented with 10 volumes % of fetal bovine serum (FBS, Sigma-Aldrich, Germany) and 1 volume % of penicillin–streptomycin (PS, Gibco, Paisley, UK) and incubated at 37 °C under a humidified atmosphere with 5% CO_2_ and 95% air. Once the MG-63 cells reached confluency, they were seeded on 24 multi-well plates with an inoculum ratio of 100,000 cells per well plus 1 mL of DMEM in each well and incubated for 24 h inside the humidified chamber. Separately, the sterilization of the MBG powders was carried out by heat treatment at 160 °C for 2 h. Indirect cytocompatibility tests were performed using extracts of MBG powders. For that purpose, 800 mg of sterilized powders were soaked in 8 mL of DMEM (10% w/v) and incubated at 37 °C for 24 h inside the humidified chamber. After 24 h, the extract was centrifuged at 4000 rpm for 15 rpm and filtered using 0.45 µm pore size filter to remove any solid particles. Then, the filtered extract was diluted to 5, 1, 0.1% w/v using DMEM. After 24 h, the cell media from the multi-well were removed, and 1 mL of the appropriately diluted extract (i.e., 10, 5, 1, 0.1% w/v) was added to the well plates and placed inside the incubator for 48 h. The cells in the DMEM without extract were used as a positive control, and the cells in (6% v/v) of dimethyl sulfoxide (DMSO) and DMEM were used as the negative control. Each experiment was performed in triplicate.

The cell viability test was performed using a standard colorimetric WST-8 assay (CCK-8, Sigma Aldrich), which is a tetrazolium salt reduced by cellular dehydrogenase enzyme to form an orange color formazan product. The amount of formazan formed is directly related to the number of living cells. After 48 h of culturing, the MG-63 cells with extract 400 µL of 1% v/v, WST-8 in colorless DMEM was added to 24-well plates and incubated for 3 h. The prepared solution of WST-8 in DMEM was also incubated for 3 h to use as a blank. Afterwards, 100 µL of aliquots were transferred from each well to 96-well plates to measure the absorbance at 450 nm from the microplate reader ((PHOmo Elisa reader, Autobio Diagnostic Co. Ltd., Zhengzhou, China). The MG-63 cells viability percentage was calculated by the following equation:

$$Cell\;viablity\;\left(\%\right)=\frac{Absorbance\;of\;sample-Absorbance\;of\;blank}{Absorbance\;of\;positive\;control-Absorbance\;of\;blank}\times 100$$

Hematoxylin and Eosin (H& E) staining was performed to view the cell morphology. The hematoxylin binds with the nuclei (DNA/RNA) of the cells and turns a dark blue color, while eosin stains the cytoplasmic filaments and extracellular matrix of the cells and turns to pink or red color. The H&E staining was performed on the same 24-well plates after the indirect cell viability test. The leftover WST solution was discarded, washed with 1 mL/well of phosphate buffer saline (PBS) and fixed with a 400 µL/well fluorescence fixer which is a 4% solution of paraformaldehyde in PBS. After 15 min, the fixation solution was removed and washed with distilled water and stained with hematoxylin (400 µL/well) for 15 min. Later, the plates were washed with distilled water, Scott’s tap water (pH 8.11 for 5 min.) and again with distilled water. Afterwards, the cells were stained with eosin solution (0.4% eosin in 60% ethanol+ 5% acetic acid + 35% distilled water) for 5 min, washed with 95% and 99.5% ethanol and dried at room temperature. Finally, the morphology of the MG-63 cells was analyzed using an optical microscope (Primo vert, Carl Zeiss).

### 4.6. Antibacterial Assessment

The antibacterial activity of MBG was evaluated against Gram-negative *Escherichia coli* (*E. coli*) and Gram-positive *Staphylococcus aureus* (*S. aureus*) bacteria by the modified agar disk diffusion method. The MBG pellets were prepared (with a dimension of ~14 mm diameter and ~2 mm thickness) using 200 mg of powders by a hydraulic press (PE-010, Mauthe Maschinenbau) with a 3-ton load and sterilized at 160 °C for 2 h.

*E. coli* and *S. aureus* bacteria suspensions were grown in lysogeny broth (LB medium) at 37 °C for 24 h. Next, 20 mL of the hot LB agar medium was poured into the Petri plates, and once the agar medium solidified, 20 µL of each bacterial suspension was spread on the top of the agar plates. Then sterilized MBG pellets were placed in the middle of the fresh bacterial culture plates and incubated overnight at 37 °C. The next day, the growth of the bacteria and the zone of inhibition surrounding the MBG pellets were determined by digital images and measured using a ruler scale in millimeters (mm).

### 4.7. Statistical Analysis

Statistical analysis was carried out using one-way ANOVA and Tukey tests. The analysis was performed using OriginLab 2018 software. The statistically significant differences were evaluated using probability (P) values *p* < 0.05. The results included the mean ± standard deviation (SD).

## Figures and Tables

**Table 1:** Represents the composition of prepared MBG powders determined by the ICP-OES technique.

Actual Compositions					
Sample	SiO_2_ (mol.%)	CaO (mol.%)	P_2_O_5_ (mol.%)	SrO (mol.%)	CuO (mol.%)
80G	84.9 ± 0.4	14.8 ± 0.4	0.28 ± 0.01	-	-
1CS	86.4 ± 0.2	12.2 ± 02	0.41 ± 0.03	0.51 ± 0.01	0.5 ± 0.01
2CS	83.8 ± 0.1	13.4 ± 0.3	0.31 ± 0.02	1.1 ± 0.02	1.2 ± 0.01
4CS	81.8 ± 0.1	12.6 ± 0.05	0.8 ± 0.06	2.2 ± 0.01	2.4 ± 0.01

**Table 2:** The textural properties of MBG (S_BET_: specific surface area, V_P_: pore volume and D_P_: pore diameter.

Sample	S_BET_ (m^2^/g)	V_P_ (cm^3^/g)	D_P_ (nm)
80G	253	0.33	5.20
1CS	348	0.44	5.06
2CS	378	0.45	4.80
4CS	332	0.39	4.71

**Table 3:** The diameters of the zone of inhibition for all MBG samples.

Sample	Zone of Inhibition (mm)	
	*E. coli*	*S. aureus*
80G	-	-
1CS	~1.5	~2
2CS	~2.3	~2.5
4CS	~3.5	~3.5

**Table 4:** The theoretical (nominal) compositions of the glass (in mol.%) and the acronyms used to indicate them.

Acronym	CaO	CuO	SrO	SiO_2_	P_2_O_5_
80G	15	0	0	80	5
1CS	14	0.5	0.5	80	5
2CS	13	1	1	80	5
4CS	11	2	2	80	5

## Data Availability

Not applicable.
